# Role of extracellular vesicles in nonalcoholic fatty liver disease

**DOI:** 10.3389/fendo.2023.1196831

**Published:** 2023-07-18

**Authors:** Wei Jiang, Youhui Xu, Jou-Chen Chen, Yi-Hung Lee, Yushin Hu, Chang-Hai Liu, Enqiang Chen, Hong Tang, Hua Zhang, Dongbo Wu

**Affiliations:** ^1^Center of Infectious Diseases, West China Hospital, Sichuan University, Chengdu, China; ^2^West China School of Medicine, Sichuan University, Chengdu, China; ^3^West China College of Stomatology, Sichuan University, Chengdu, China; ^4^Key Laboratory of Birth Defects and Related Diseases of Women and Children of MOE, State Key Laboratory of Biotherapy, West China Second University Hospital, Sichuan University, Chengdu, China; ^5^NHC Key Laboratory of Chronobiology, Sichuan University, Chengdu, China; ^6^Sichuan Birth Defects Clinical Research Center, West China Second University Hospital, Sichuan University, Chengdu, China

**Keywords:** extracellular vesicles (EV), NAFLD, diagnosis, treatment, mechanisms

## Abstract

**Background:**

Nonalcoholic fatty liver disease (NAFLD) is a common chronic liver disease that affects approximately one-quarter of the global population and is becoming increasingly prevalent worldwide. The lack of current noninvasive tools and efficient treatment is recognized as a significant barrier to the clinical management of these conditions. Extracellular vesicles (EVs) are nanoscale vesicles released by various cells and deliver bioactive molecules to target cells, thereby mediating various processes, including the development of NAFLD.

**Scope of review:**

There is still a long way to actualize the application of EVs in NAFLD diagnosis and treatment. Herein, we summarize the roles of EVs in NAFLD and highlight their prospects for clinical application as a novel noninvasive diagnostic tool as well as a promising therapy for NAFLD, owing to their unique physiochemical characteristics. We summarize the literatures on the mechanisms by which EVs act as mediators of intercellular communication by regulating metabolism, insulin resistance, inflammation, immune response, intestinal microecology, and fibrosis in NAFLD. We also discuss future challenges that must be resolved to improve the therapeutic potential of EVs.

**Major conclusions:**

The levels and contents of EVs change dynamically at different stages of diseases and this phenomenon may be exploited for establishing sensitive stage-specific markers. EVs also have high application potential as drug delivery systems with low immunogenicity and high biocompatibility and can be easily engineered. Research on the mechanisms and clinical applications of EVs in NAFLD is in its initial phase and the applicability of EVs in NAFLD diagnosis and treatment is expected to grow with technological progress.

## 1 Introduction

Nonalcoholic fatty liver disease (NAFLD) has become the most common chronic liver disorder affecting approximately 25% of the global adult population, which prevalence varies from 13.5% in Africa to 31.8% in the Middle East (1), causing a growing global burden of liver diseases (2). NAFLD encompasses a disease continuum from nonalcoholic fatty liver (NAFL) to nonalcoholic steatohepatitis (NASH), which is characterized by necroinflammation and faster fibrosis progression than NAFL (3). Patients with NAFLD are prone to developing cirrhosis and hepatocellular carcinoma (HCC), making NAFLD the most rapidly growing cause of liver transplantation in HCC patients, with an 11.8-fold increase during 2002-2016 (4, 5). NAFLD has become the most rapidly growing contributor to liver mortality and morbidity (6). Drugs such as glucagon-like peptide-1 (GLP-1) agonists, pioglitazone, and sodium-dependent glucose transporter 2 (SGLT2) inhibitors are currently available for the treatment of obesity and type 2 diabetes mellitus(T2DM), but there is currently no FDA-approved drug therapy for NASH (3). A healthy lifestyle and weight management remain central to the prevention and treatment of NAFLD (7).

The pathophysiology of NASH is multifactorial, involving genetic and epigenetic factors, insulin resistance (IR), adipose-derived hormones, over-nutrition, and microbiome-related factors that are not well understood, and several studies have reported that extracellular vesicles (EVs) play a significant role in the development of NAFLD (3, 8, 9). EVs act as intercellular mediators and participate in metabolic regulation, inflammatory and immune responses, intestinal microecological balance, and fibrotic processes. Therefore, understanding the mechanism of EVs is of great significance for improving the diagnosis and treatment of NAFLD. In this review, we summarize the roles of EVs in NAFLD and highlight their utility as diagnostic and therapeutic tools in NAFLD.

## 2 The characteristic of extracellular vesicles

**Figure 1:**
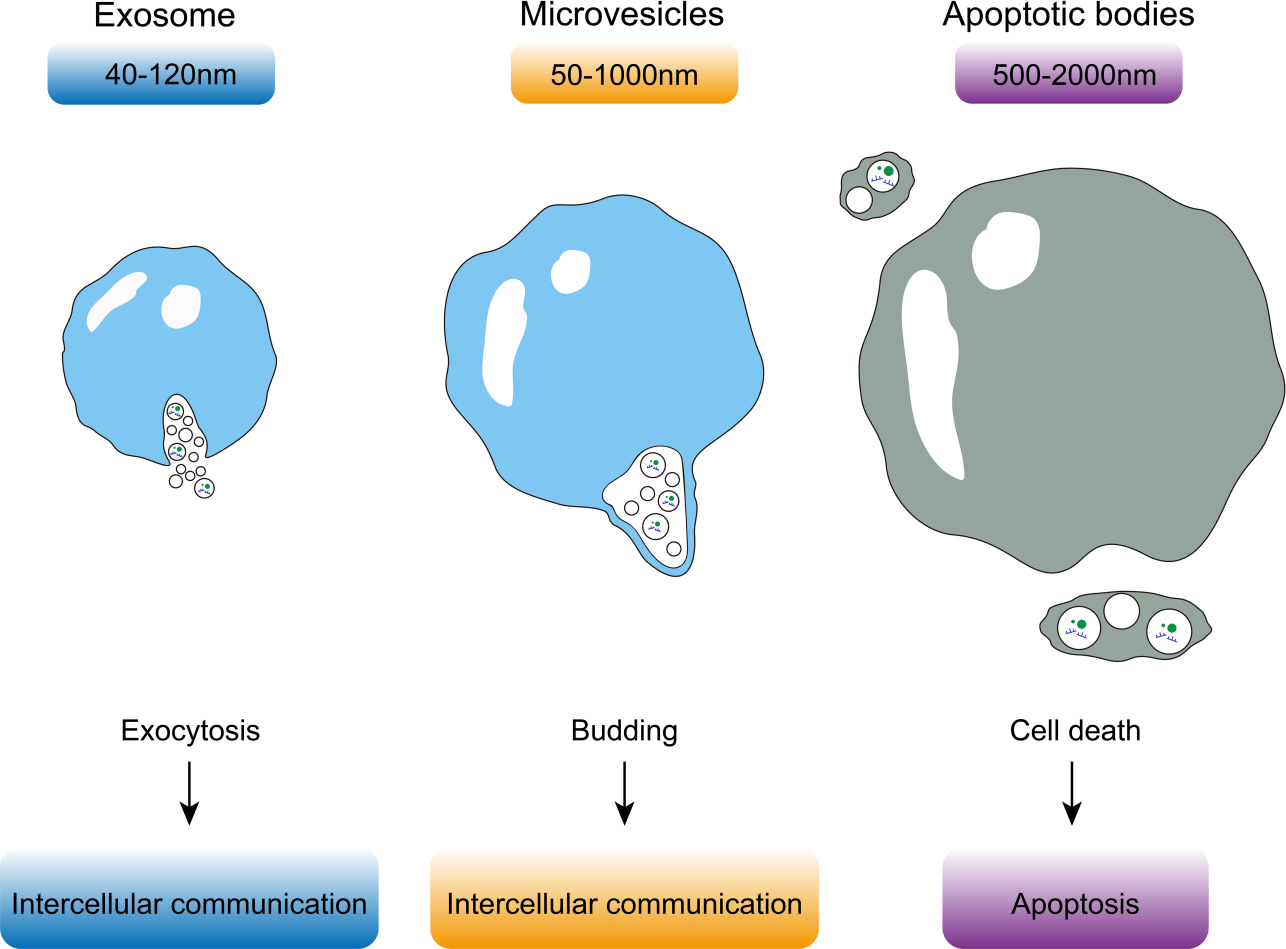
Exosomes are the smallest EVs with a diameter of 40-120 nm and are formed due to exocytosis. Microvesicles are derived from cellular budding and have a larger size of 50-1000 nm. Apoptotic bodies are always derived from dead cells and are the largest of EVs with a size of 500-2000 nm.

An EV is a membranous vesicle derived from the cellular membrane systems of living cells with lipid bilayer membranes (10). EVs were first observed in plasma in 1967 (11). Since then, it has been established that EVs can be isolated from a variety of body fluids (12–16) and be released by almost all types of cells (17–20). Various proteins, lipids, DNA, RNA, and metabolic products, which have been proven to regulate gene expression and signaling pathways in cells, can function as cargo for EVs (21, 22). The biosynthesis of EVs may be regulated by EV cargoes bind trafficking effectors, which enrich cargoes in endosomal and plasma membrane patches, and cause the endosomal membrane to bud into the lumen of the endosome, leading to the formation of intraluminal vesicles as early endosomes mature into late endosomal multivesicular body (23). Depending on their source, size, and function, EVs can be divided into three types—exosomes, microvesicles, and apoptotic bodies (**Figure 1**) (24, 25). Exosomes are the smallest EVs with a diameter of 40-120 nm and are formed due to exocytosis. Microvesicles are derived from cellular budding and have a larger size of 50-1000 nm. Both exosomes and microvesicles play a role in intercellular communication. Apoptotic bodies are always derived from dead cells and are the largest of EVs with a size of 500-2000 nm. Different surface modifications of EVs, such as exosomes, can make it have different functions, so as to achieve targeted drug delivery and *in vivo* imaging and tracking. Specific membrane proteins functionalized on the surface of exosomes, such as tetraspanins proteins (CD63, CD81, CD9), lactadherin, lysosome-associated membrane protein-2B, and glycosyl phosphatidylinositol, as well as different surface modification strategies such as genetic engineering, interact with the receptor system of target cells, which is involved in the regulation of physiological functions of various organ systems (26).

## 3 The role of EVs in NAFLD

**Figure 2:**
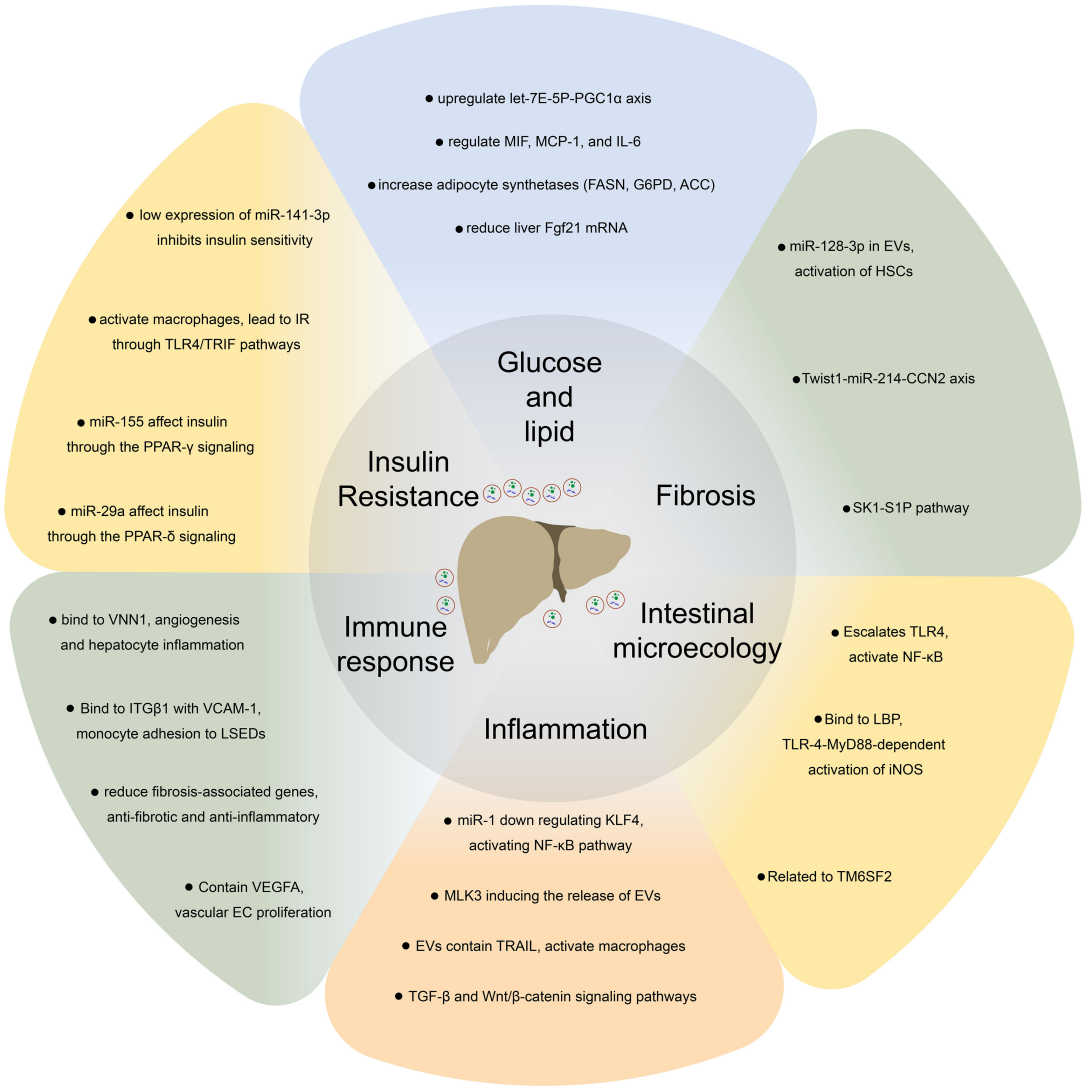
EVs regulate glucose and lipid metabolism, fibrosis, intestinal microecology, inflammation, immune response, insulin resistance and other processes by recognizing target cells and binding to cell-specific receptors or fusion with the target cell membrane to achieve substance transport and induce intracellular signal transduction, thus, participate in the pathophysiological process of non-alcoholic fatty liver disease.

EVs contain specific molecules on their surface that can induce signal transduction in specific cells by recognizing target cells and binding to cell-specific receptors or fusing with the target cell membrane and transferring the cargo into their cytoplasm to regulate the physiological activities of cells (**Figure 2**) (21). Based on different cell sources, EVs participate not only in normal physiological processes but also in disease processes (27–29). Consequently, EVs have potential application value as diagnostic biomarkers (30). In the liver, EVs not only play an important role in mediating signal transduction in liver cells but also affect metabolic pathways in liver cells associated with apoptosis of hepatocytes, inflammation, liver fibrosis, and the development of NAFLD (**Table 1**) (55, 56).

**Table 1:** Mechanisms of development of nonalcoholic fatty liver disease related to extracellular vesicles.

Source of EVs	Mechanism	Pathophysiological progress		Reference
Glucose and lipid				
		Glucose metabolism	Lipid metabolism	
Hepatocytes	EVs containing let-7e-5p enhances adipocyte lipid deposition through Pgc1α.		↑	Yue Zhao, etal. (31)
Adipocytes	Hypoxic adipocyte-released exosomes were enriched in enzymes related to *de novo* lipogenesis (FASN, G6PD, ACC)		↑	Soichi Sano, etal. (32)
Body fluids	Obesity-associated exosomal miRNAs (miR-192 and miR-122) induce glucose intolerance and dyslipidemia	↑	↑	Carlos Castaño etal. (33)
HASCs	Secreted EVs during stem cell differentiation into white adipocytes or beige adipocytes can promote cell reprogramming.		↓	Youn Jae Jung, etal. (34)
IR				
		Insulin sensitivity	IR	
Plasma	IR increases the secretion of EVs, which are preferentially internalized by leukocytes, and alters leukocyte function.		↑	David W Freeman, etal. (35)
adipose tissue	Exosomes released from obesity adipose tissue containing less miR-141-3p inhibit the insulin sensitivity and glucose uptake.		↑	Shi-Ying Dang, etal. (36)
adipose tissue	The ob-EVs mediate the induction of TNF-α and IL-6 in macrophages and IR through the TLR4/TRIF pathway		↑	Zhong-bin Deng, etal. (37)
ATMs	Exosomes from ATMs in obese mice containing miR-155 cause glucose tolerance and IR by targeting PPARγ.		↑	Wei Ying, etal. (38)
Skeletal muscles	Exosomes from skeletal muscles of IUGR containing miR-29a induce IR though PPARδ/PGC-1α−dependent signals.		↑	Yuehua Zhou, etal. (39)
Immune response				
		Anti-inflammation	Pro-inflammation	
Hepatocytes	Lipotoxic hepatocyte-derived EVs are enriched with active ITGβ1, which promotes monocyte adhesion and liver inflammation in murine NASH.		↑	Qianqian Guo, etal. (40)
Hepatocytes	Lipids-induce-released hepatocyte EVs activate an inflammatory phenotype in macrophages by stimulating DR5.		↑	Petra Hirsova, etal. (41)
Hepatocytes	Cholesterol-induced lysosomal dysfunction increases the release of exosome containing miR-122-5p from hepatocytes, resulting in M1 polarization and macrophage-induced inflammation.		↑	Zhibo Zhao, etal. (42)
Hepatocytes	Steatotic hepatocyte-derived EVs promote endothelial inflammation and facilitate atherogenesis by miR-1 delivery, KLF4 suppression and NF-κB activation		↑	Fangjie Jiang, etal (43).
Neutrophils	miR-223-enriched EVs derived from neutrophils acted to inhibit hepatic inflammation and fibrosis	↑		Yong He, etal (44)
Hepatocytes	MLK3 mediates the release of CXCL10-laden EVs from lipotoxic hepatocytes, which induce macrophage chemotaxis		↑	Samar H Ibrahim, etal. (45)
Intestinal microecology				
		remission	aggravate	
Bacteria	LPS and palmitate induce the expression of TLR4 and NF-κB to promote NASH.		↑	Torfay Sharifnia, etal. (46)
Bacteria	LPS can promote the decrease of plasma adiponectin, the increase of plasma leptin levels, and greater expression of FAS and SREBP-1c mRNA in the liver		↑	Shinya Fukunishi, etal. (47)
Bacteria	The loss of functional LBP protected against early stages of NAFLD development, in part due to the protective effect of TLR-4–MyD88-dependent iNOS activation.		↑	Cheng Jun Jin, etal. (48)
Liver fibrosis				
		Anti- fibrosis	Pro-fibrosis	
EC	EC-derived SK1-containing exosomes regulate HSC signaling and migration through FN-integrin-dependent exosome adherence and dynamin-dependent exosome internalization		↑	Ruisi Wang, etal. (49)
HSCs	Cellular or exosomal Twist1 drives miR-214 expression and suppresses CCN2 production and downstream fibrogenic signaling through transcriptional activation of the DNM3os E-box	↑		Li Chen, etal. (50)
HLSC	HLSC-derived EVs attenuate liver fibrosis and inflammation		↑	Stefania Bruno, etal. (51)
Hepatocytes	Lipotoxic hepatocyte-derived EVs containing miR128-3p inhibit PPAR-γ to activate HSCs.		↑	Davide Povero, etal. (52)
PMFs	PMFs released VEGFA-containing microparticles, which activated VEGF receptor 2 in ECs and largely mediated their proangiogenic effect.		↑	Sara Lemoinne, etal. (53)
Hepatocytes	Exosomes secreted by hepatocytes exposed to FFA contribute to angiogenesis and liver damage in steatohepatitis requiring VNN1-dependent internalization		↑	Davide Povero, etal. (54)

IR, insulin resistance; IUGR, intrauterine growth retardation; EVs, extracellular vesicles; FASN, fatty acid synthase; G6PD, 6-phosphate-glucose dehydrogenase; ACC, 1.acetyl-CoA carboxylase; HASCs, human adipose-derived stem cells; TNF-α, tumor necrosis factor-α; IL-6, interleukin-6; TLR4/TRIF, toll-like receptor 4/TIR domain-containing adaptor protein inducing interferon; ATMs, adipose tissue macrophages; PPARγ, peroxisome proliferator−activated receptor γ; PPARδ, peroxisome proliferator−activated receptor δ; PGC-1α, proliferator-activated receptor-γ coactivator-1α; PMFs, portal myofibroblasts; VEGF, vascular endothelial growth factor; ECs, endothelial cells; VNN1, Vanin-1; ITGβ1, integrin β1; DR5, death receptor 5; KLF4, Kruppel like factor 4; NF-κB, Nuclear Factor-κB; HSCs, hepatic stellate cells; HLSC, human liver stem cells; MLK3, mixed lineage kinase 3; CXCL10, (C-X-C motif) ligand 10; SK1, sphingosine kinase 1; FN, fibronectin; LBP, lipopolysaccharide-binding protein; iNOS, inducible nitric oxide synthase.

### 3.1 Glucose and lipid metabolism

Patients with NAFLD often have glucose and lipid metabolic disorders, which have been proven to be regulated by EVs and are associated with multiple pathways (57–59). Some research report that obese individuals have higher levels of circulating EVs than normal-weight individuals (60, 61). While, the role of EVs in regulating the glucose and lipid metabolism is multifarious. The possible mechanism may be the diversity of contents carried by exosomes, including non-coding RNAs and cytokines, which play biological functions.

A recent report indicated that adipocyte-derived EVs may induce hepatitis and cirrhosis by regulating adipose tissue homeostasis, interfering with normal signaling pathways, and causing metabolic dysfunction (22). EVs play a significant role in lipid redistribution in metabolic organs such as the liver, adipose tissues, and muscles under lipid overload. The study found that EVs levels increased in response to acute lipid overload and these EVs containing let-7e-5p fuse with adipocytes to promote adipocyte regeneration by upregulating the let-7E-5p-PGC1α axis (31). In a relatively hypoxic environment, the secretion of EVs derived from 3T3-L1 adipocytes increased. Proteomic analysis revealed 231 protein components in these EVs, including a variety of lipogenic enzymes such as fatty acid synthase (FASN), 6-phosphate-glucose dehydrogenase (G6PD), and acetyl-CoA carboxylase (ACC), which promote fat synthesis and increase adipocyte load (32). Adipocyte-derived EVs act as adipocytokines that regulate the secretion of cytokines from adjoining cells in response to a variety of stimuli. They activate macrophages and promote the synthesis and release of macrophage colony-stimulating factor, interleukin-6 (IL-6), and tumor necrosis factor-α (TNF-α), which aggravate IR, destroy gluconeogenesis in liver tissue and promote liver inflammation by adjusting the release of macrophage migration inhibitory factor (MIF), macrophage chemoattractant protein-1 (MCP-1) and IL-6 (62). EVs isolated from human adipose-derived stem cells (HASCs) generated during beige adipogenic differentiation can differentiate HASCs into beige and brown adipocytes. EVs derived from beige/brown adipocytes have beneficial effects on the browning of the white adipose tissue (22, 34). A study by Thomou et al. (63) showed that circulating EVs isolated from adipose tissue-specific miRNA knockout mice contain decreased miRNAs, and circulating miRNA levels are almost completely restored after transplantation of white/brown adipose tissue. These miRNAs play a role in improving glucose tolerance and reducing fibroblast growth factor 21(Fgf21) mRNA in hepatocytes. Another study showed that EVs containing let-7b-5p activated TGF-β-let-7b-5p signaling pathway in hepatocytes, reducing mitochondrial oxidative phosphorylation and suppressing white-to-beige fat conversion, that promoted high-fat diet (HFD)-induced steatosis and obesity (64). In summary, EVs can regulate glucose and lipid metabolism and NAFLD development by regulating gene expression or cell-specific signaling pathways. By blocking specific signaling pathways in or receptors on target cells, we can modulate the effects of EV cargo. The findings of the studies discussed above may provide new insights for the research and development of novel drugs in the future.

### 3.2 Insulin resistance

IR is closely related to liver steatosis and can also predict the development of NAFLD (65). Recent studies have revealed that EVs are easily internalized by cells and cause functional changes in specific tissues, regulating insulin signaling in other tissues (35). Adipocyte-derived exosomes can cause IR. The first study that identified the role of adipocyte-EVs in IR was performed in a mice model of obesity (37). EVs released by adipocytes can activate macrophages, which can induce IR through toll-like receptor 4/TIR domain-containing adaptor protein inducing interferon-β (TLR4/TRIF) pathways (37). Dang et al. (36) proposed that IR in obese individuals is highly correlated with the low expression of miR-141-3p in exosomes secreted by adipose tissues. EVs released by adipose tissue from obese mouse models can mediate crosstalk between adipose tissues and macrophages. Additionally, some studies have suggested that insulin sensitivity is related to macrophages that reside within adipose tissue (ATMs). Another study on ATMs found that miR-155 in exosomes released by ATMs was overexpressed in an obese mouse model (66). And it is reported that miR-29a was overexpressed in exosomes derived from ATMs in obese mouse models (38). miR-155 and miR-29a are key mediators s in the peroxisome proliferation-activated receptor-γ (PPAR-γ) and PPAR-δ signaling pathways, respectively. Both PPAR-γ and PPAR-δ have been identified as targets of miRNAs that regulate IR (39). The studies suggest that ATMs can impair insulin sensitivity by secreting exosomes containing specific miRNAs, leading to the inhibition of glucose uptake and directly affecting the insulin levels in an organism. Anja Fuchs et al. (67) found that systemic IR in people with obesity and NAFLD is associated with increased plasma PAI-1 concentrations and both plasma and subcutaneous abdominal adipose tissue derived exosomes. In addition, gut microbial-derived EVs can also influence glucose metabolism by regulating IR (68). It was found that fecal-derived EVs induced IR and poor glucose tolerance in high-fat diet (HFD)-fed mice compared to conventional diet-fed mice (69). In summary, EVs have been implicated in the development of IR and understanding the molecular mechanisms by which they confer IR may be effective in the prevention or treatment of NAFLD.

### 3.3 Immune response

The persistent inflammatory response is an important cause of the transition from simple fatty liver disease to severe liver injury, such as steatohepatitis and cirrhosis, which is related to the immune response (70). Immune regulation, including innate and adaptive immunity, is crucial to the pathogenesis of NAFLD. Innate immune cells in the liver include Kupffer cells, dendritic cells, natural killer (NK) cells, innate lymphoid cells, invariant NKT cells, and mucosal-associated invariant T cells, which form the first line of defense against invading organisms and environmental challenges. The hepatic innate immune response plays a prominent role in the progression of liver disease; therefore, it is an important driving force in NAFLD (71). Increasing evidence suggests the role of lymphocyte-mediated adaptive immunity as a factor promoting liver inflammation, including the role of B cells and CD4^+^T and CD8^+^T cells in sustaining NASH progression (72).

#### 3.3.1 Innate immunity

Patients with NAFLD show increased levels of EVs derived from macrophages and NK cells. The levels of EVs derived from immune cells can be used to assess the extent of chronic liver disease, which is related to the enhancement of innate immune function during the development of NAFLD (73). EVs promote pathological angiogenesis and fibrosis in NASH by transporting a variety of mediators including growth factors, hedgehog molecules, proteins, and miRNAs (74). A study by Pover et al. (54) showed that under saturated lipotoxicity, the caspase8-caspase3-ROCK1 pathway in hepatocytes is activated, releasing EVs containing a large amount of Vanin-1, which can reinforce the internalization of EVs, initiate the migration of endothelial cells (ECs), and promote the generation of new small blood vessels, resulting in hepatocyte inflammation. Lemoinne et al. (53) showed that EVs carrying vascular endothelial growth factor A (VEGF-A) can be released by activated portal myofibroblasts and bind to VEGF-A receptors on vascular EC to promote vascular ECs and ductal hyperplasia. Vascular ECs co-cultured with steatotic hepatocytes or treated with steatotic hepatocyte-derived EVs decreased Kruppel-like factor 4 release, which activated the intracellular NF-κB pathway and significantly increased pro-inflammatory factor release (43).

Lipotoxic liver cells can release EVs that contain various macrophage chemokines and active mediators. Protein mass spectrometry of EVs showed that EVs contain many damage-associated molecular patterns, which can activate the inflammatory response in mammals (75). Lipid molecules can promote NAFLD by activating cytokines in hepatocytes. To activate the death receptor 5 (DR5) of hepatocytes through non-ligand-dependent pathways, the secretion of EVs in hepatocytes increase. TNF-related apoptosis-inducing ligand (TRAIL) on the surface of the EVs activates DR5-RIP1-NF-κB signaling pathway in macrophages to increase the secretion of IL-1β and IL-6 (41), which aggravates inflammation in hepatocytes. EVs carrying miR-122-5p secreted by hepatocytes can stimulate the secretion of pro-inflammatory factors and polarize hepatic macrophages into the M1 phenotype (42). Studies also have reported that EVs can mediated the macrophages, which is supposed to play an important role in the regulation of fibrosis (76, 77). Several studies have demonstrated that NAFLD are associated with exosomes derived from or transferred to macrophages (78). For example, exosomal miRNA-411-5p derived from M2 macrophages plays an inhibitory role in HSCs activation during NASH progression by inhibiting its target gene CAMSAP1 (79). Furthermore, the exosomes released by lipotoxic hepatocytes can be ingested by macrophages, resulting in activation of M1 macrophages and hepatic inflammation by regulating the Rictor/Akt/FoxO1 signaling pathway (80). Hepatocyte-derived EVs can also promote monocyte adhesion via an integrin β1-dependent mechanism to induce an inflammatory response (40). Besides, under the inflammation or mechanical stimulation, the hepatic stellate cells (HSCs) activated and participate in the formation of liver fibrosis through the proliferation and secretion of the extracellular matrix. One possible mechanism for this transformation is through the upregulation and release of miR128-3p by EVs under lipotoxicity caused by increased free fatty acids in hepatocytes (64). These hepatocyte-derived EVs are internalized by HSCs and inhibit PPAR-γ in quiescent HSCs to facilitate phenotypic conversion (52). When HSCs were exposed to miR128-3p-deficient EVs, a higher PPAR-γ level and reduced proliferation and migration were observed. EVs containing connective tissue growth factor or miR214 promote the phenotypic transformation of activated HSCs (81). This is a possible mechanism of the translation from NAFLD to liver fibrosis and NASH. However, some studies suggest that EVs derived from hepatocytes can significantly downregulate the expression of genes related to fibrosis and have anti-inflammatory and anti-fibrotic effects. Neutrophil-derived miR-223 with high apolipoprotein E expression can be taken up by hepatocytes to limit the progression of steatosis to NASH (44). Fibrosis-related genes were significantly downregulated in immune-deficient NASH mice (methionine-choline-deficient diet-induced) that were treated with human hepatocyte-derived EVs (51). EVs from human liver stem cells are believed to slow down the symptoms of fibrosis and inflammation by regulating gene expression in liver cells. Therefore, the role of EVs in innate immunity appears to be dynamic and must be further investigated.

#### 3.3.2 Adaptive immunity

The current concept is that innate immunity represents a key element in development of NAFLD, however, adaptive immunity is increasingly being recognized as an additional factor of NAFLD (72). NASH is characterized by increased levels of liver and circulating IFN-γ-producing CD4^+^ T cells (82). CD4^+^ T cells can differentiate into T helper 17 cells that release IL-17. IL-17 can promote M1-type macrophage polarization and exacerbate the liver inflammatory response to accelerate NAFLD progression (83). Mice lacking CD8^+^ T cells and NKT cells are protected from steatosis and NASH when fed with a choline-deficient HFD, which is associated with reduced production of LIGHT by CD8^+^T cells and NKT cells (84). Adaptive immunity and innate immunity are not completely independent, and there is an interplay between the two. Sun et al. (85) showed that OX40 was a key regulator of intrahepatic innate and adaptive immunity and mediated two-way signals and promotes both pro-inflammatory monocytes and macrophages, as well as T cell function, resulting in the development of NASH. By promoting NK cell activation, lymphocytes stimulate the secretion of IL-15 and IL-18 by macrophages, thereby modulating the progression of steatohepatitis and fibrogenesis (86). In conclusion, adaptive immune responses are crucial in the progression of NAFLD. EVs have been proven to be key factors in mediating adaptive immune responses by playing roles in antigen presentation, T-cell activation, T-cell polarization to regulatory T-cells, and immune suppression (87). Therefore, EVs play a role in NAFLD through the modulation of adaptive immunity.

### 3.4 Inflammation

Recently, many studies have found that EV levels significantly increase in NASH mice models (54, 75). HFD promotes the release of EVs, and the number of EVs increases in a time-dependent manner (88). MiR-1 in hepatocyte-derived EVs is an important factor in the promotion of endothelial inflammation. EVs aggravate not only endothelial inflammation but also atherosclerosis by delivering miR-1, which induces the inhibition of Kruppel like factor 4 (KLF4) and activation of the NF-κB pathway (43). In addition to promoting inflammation in endothelial cells, EVs also mediate inflammation through macrophages. Several studies have shown that the aggregation of Kupffer cells is closely related to hepatocyte-derived EV levels (75, 89–91), suggesting that EVs mediate the inflammatory response in liver damage by inducing chemotaxis of macrophages. In lipotoxic hepatocytes, the activated mixed lineage kinase 3 pathway promotes EV secretion by upregulating c-Jun N-terminal kinase. The secreted EVs further mediate chemotaxis of macrophages by releasing C-X-C motif ligand 10 (CXCL10) via binding to C-X-C receptor-3 (CXCR-3) and promoting macrophage-associated hepatic inflammation (45). Garcia-Martinez, et al. (92) found higher levels of Mitochondrial DNA (mtDNA) in EVs of mice and patients with NASH, with concurrent increase in hepatocyte-specific marker that activate toll-like receptor 9 (TLR9). TLR9 can mediate inflammation, thereby contributing to the transition from simple steatosis to steatohepatitis. Another study has shown that the mechanism of released EVs is related to the activation of the DR5 signaling pathway and the activation of macrophages by TRAIL of the released EVs to promote a metabolic response (41). Ferrante et al. (93) analyzed EVs shed by adipocytes from obese people and confirmed that adipocyte-derived EVs participate in transforming growth factor (TGF)-β and Wnt/β-catenin signaling pathways through miRNAs, which promote inflammation and fibrosis. In summary, the lipotoxicity in hepatocytes promotes the release of EVs, and increased EVs mediate the inflammatory response by enabling intercellular interaction.

### 3.5 Intestinal microecology

Intestinal microorganisms produce a variety of proteins and bile acids, participate in bidirectional communication along the enterohepatic axis, and regulate intestinal microecology. Damage to gut microflora balance, such as changes in intestinal microflora composition and intestinal bacterial metabolites, plays an important role in regulating the development of NAFLD (58). Many bacteria-derived molecules, including nucleic acids, proteins, polysaccharides, and glycolipids, exist in microbe-derived EVs (21). These EVs not only support the survival of bacteria by delivering virulence factors and nutrients but also participate in the regulation of multiple signaling pathways in host cells (94). They influence NAFLD by regulating glucose and fat metabolism, immune responses, and redox balance (95).

Bacterial EVs can trigger multiple metabolic cascades and immune responses (95). Bacteria-derived EVs contain and transfer lipopolysaccharide (LPS), enter hepatocytes via the biliary tract, portal vein, and enterohepatic axis, and aggravate NAFLD. These EVs can induce liver inflammation by activating the TLR4-TRIF-GBPs signaling pathway (96) or delivering LPS into the cytosol of host cells to activate caspase-11, which regulates the immune response (97). Compared to patients with NAFLD, patients with NASH show a significant increase in LPS and free fatty acid (FFA), as well as an increase in TLR4 mRNA and interferon regulatory factor 3 (IRF-3) in the myeloid differentiation factor 88-independent signaling pathway. In addition, when using small interfering RNA-mediated TLR4 inhibitors, the inductive effect of LPS on NF-κB was weakened, suggesting that LPS can affect the TLR4-mediated NF-κB signaling pathway (46). TLR4 activates downstream signaling pathways that stimulate the release of cytokines and chemokines, leading to liver damage (47).

LPS-binding protein (LBP) and CD14 also participate in recognizing LPS, which is increased in NASH and NAFLD patients. LBP knockout in mice and subsequent prevention of LPS and TLR4 binding improved lipid metabolism in mice, protecting them from developing NAFLD under HFD conditions (48), suggesting that LBP is a crucial factor in NAFLD development. LBP and LPS levels have been shown to be associated with the development of NASH and fibrosis (98). Short RNA (sRNA) from bacteria-derived EVs can participate in regulating the innate immune response in host animals (99). Thus, damage to intestinal microecology can promote NAFLD by regulating intestinal bacteria-derived EVs containing sRNA or LPS.

As discussed above, gut microbiota-derived EVs may affect NAFLD through different mechanisms. Therefore, augmentation of beneficial gut microbes is a potential therapeutic approach. Previous studies have found that probiotics, prebiotics and other products can improve the condition of NAFLD patients (100). For example, ingestion of Lactobacillus acidophilus La5 and Bifidobacterium lactis Bb12 improved liver enzyme, serum total cholesterol, and LDL cholesterol levels in patients with NAFLD (101). Prebiotics significantly reduced TNF-α, CRP, liver enzymes, and steatosis in patients (102). Considering the potential benefits of probiotic transplantation and prebiotics in the treatment of NAFLD, combined with the role of EVs in NAFLD, we believe that this will be a direction of great research potential in the future, but further in-depth research is still needed.

### 3.6 Liver fibrosis

In addition to promoting inflammation, EV levels can influence liver fibrosis. Studies have shown that EVs can induce the activation of HSCs (52, 56, 103) and transmit information between liver cells and HSCs (104). Many studies have confirmed that HSC activation and proliferation are closely related to liver fibrosis (105). A study showed that miR-128-3p in EVs plays a crucial role in HSC activation, indicating that hepatocyte-derived EVs can mediate HSC activation through endocytosis (52). Additionally, HSCs can deliver connective tissue growth factor (CCN2) via the secretion of EVs. Besides CCN2, Twist1 and miR-214, which comprise the Twist1-miR-214-CCN2 axis in HSCs, also mediate fibrosis through delivery by EVs (50). Moreover, the migration of HSCs is affected by EC-derived EVs containing sphingosine kinase 1 (SK1), which mediates HSC chemotaxis through the SK1-S1P pathway (49). Besides, Studies have reported that PTEN has been proved to play an important role in the fibrosis in kidney (106) and liver (107) and highly related to exosome. For example, lipotoxic hepatocytes exosome transplantation aggravated the degree of PTEN-induced expression of putative protein kinase 1 (PINK1) mediated mitophagy suppression, steatohepatitis, lipidosis, and fibrosis in the livers of NAFLD mice with cirrhosis (108). Research have found that transfer of circDIDO1 mediated by MSC-isolated exosomes can suppress HSC activation through the miR-141-3p/PTEN/AKT pathway to suppress the proliferation, reduce pro-fibrotic markers, and induce apoptosis as well as cell cycle arrest in HSCs (109). The lipotoxic hepatocyte-derived exosomal miR-1297 could promote the activation and proliferation of HSCs through the PTEN/PI3K/AKT signaling pathway, accelerating the progression of NAFLD (110). In conclusion, as mediators of communication between cells, EVs play a significant role in the development of liver fibrosis by interacting with HSCs in different ways, such as by regulating specific signaling pathways.

## 4 Applications of EVs in NAFLD

We summarized the ways in which EVs play an important role in glucose and lipid metabolism, insulin residence, immune response, inflammation, intestinal microecology, and fibrosis in NAFLD. Several studies investigating the biological mechanisms of EVs have addressed their utility in the diagnosis and treatment of complex pathologies. Owing to the complex cargo and delivery functions of EVs, they can be used as part of a multicomponent diagnostic strategy for disease detection and as a targeting vehicle for disease therapy (111). Herein, we discuss the potential diagnostic and therapeutic applications of EVs.

### 4.1 Diagnostic utility of EVs in NAFLD

There is currently no reliable method to diagnose or stage NAFLD except via invasive liver biopsy. Some studies have shown that the components in circulating EVs, such as RNAs and proteins, provide new evidence for the diagnosis of NAFLD and NASH (112), suggesting the potential of liquid biopsy as a noninvasive and accurate approach to diagnose and monitor NAFLD (113, 114). Therefore, EVs as biomarkers can be measured in body fluids and may be a promising noninvasive method for diagnosing NAFLD, overcoming some limitations of surgical biopsy (25, 112). For example, miR-135a-3p-enriched EVs have been proven to be an accurate and sensitive biomarker in NAFLD. It has been shown that the amount of circulating EVs was significantly increased after 8 weeks L-amino acid defined diet, and miRNA-122 and miR-192 are enriched in circulating EVs in NAFLD (115, 116). Therefore, the EV levels change at the early stage of NAFLD, and can be traced to identify the latent development of potential fatty liver disease at an early stage; this may be valuable for the early diagnosis of NAFLD. The contents in EVs also change dynamically at different stages during the progression of NAFLD (25) and can be exploited for identifying biomarkers for sensitively monitoring the progression of NAFLD (117). Newman et al. (118) found a stable predictive performance for total cell-free RNA and EV derived miR-128-3p in health people, NAFL and NASH patients. Therefore, EV-derived miRNA biomarkers can robustly distinguish patients with NAFL and NASH and show the severity of NAFLD.

In addition to NAFLD, EVs have been used as biomarkers in liquid biopsies for cancer diagnosis, monitoring, and prognosis (119, 120). The development of engineered EVs as individualized imaging diagnostic reagents and for facilitating targeted therapy has been proposed (121). Many exosome sensing technologies including exosome chips, EV array, and proteomic platforms, are designed to detect EVs in cancers, and CD26, CD81, and CD10 have been proposed as markers for the detection of hepatic damage associated with liver cancer (120, 122–124).

In recent years, researchers have also found that the composition of circulating exosome content in peripheral blood may be significantly changed in obese patients after bariatric surgery, and the content of circulating exosomes may be used as a serological marker to evaluate the prognosis of bariatric surgery (125–127). For example, it is reported that the microRNA content of circulating adipocyte-derived exosomes isolated from the peripheral blood are significantly modified following gastric bypass bariatric surgery and these changes are correlated to improvements in IR post-surgery (128).Another study found that total circulating EVs and hepatocyte-derived EVs are elevated in NAFLD and decrease following NAFLD resolution due to weight loss surgery, which may be new biomarkers for NAFLD resolution and response to weight loss surgery (129). In conclusion, the changes in the types and quantities of peripheral exosome contents may be used as a new indicator to evaluate the efficacy of preoperative and postoperative bariatric surgery.

### 4.2 Therapeutic utility of EVs in NAFLD

EVs have potential benefits as key mediators of cell therapy because of their advantageous features of product stability, immune tolerability, effectiveness in systemic delivery, and efficacy enhancement (130).

Currently, many studies have explored the therapeutic application of EVs (131). These include the use of mesenchymal stem cell (MSC)-derived EVs in the treatment of SARS-CoV-2-associated pneumonia (132) and the use of ticagrelor to decrease the release of procoagulant EVs from activated platelets to treat patients with myocardial infarction (133). EV-based antitumor and antibacterial vaccines have shown good safety and tolerance in patients with advanced melanoma and non-small cell lung cancer (134). Some EV cargos alleviate NAFLD.

After treatment with MSC exosomes, the levels of blood glucose and insulin, volume of visceral fat, number of lipid droplets, ballooning degeneration in liver tissue, and NAFLD activity score decreased in NASH mice. MSC exosomes can alleviate fatty liver in NASH mice and promote M2 polarization of macrophages (our unpublished data). A melanocorticosterone type 4 receptor knockout NASH mouse model challenged with LPS showed that treatment with MSC-derived EVs had anti-inflammatory and anti-fibrotic effects (135). Many studies indicate that the development of drugs to inhibit the expression of certain genes or signaling pathways with EVs participation may prevent lipid deposition and fibrosis (70, 136, 137). For example, the ROCK1 inhibitor fasudil can effectively block lipotoxicity-induced EV release in mouse models and prevent NASH progression *in vivo* (41).

As natural carriers of functional small RNA and proteins, EVs also have high application potential as drug delivery systems with low immunogenicity and high biocompatibility for chemotherapy (138, 139). In addition, EVs can be engineered to enhance bioactivity and targeting ability, avoid undesired and unnecessary cell toxicity, and enhance therapeutic effects (140). Zhang et al. found that compared with chemotherapy alone, umbilical cord-derived macrophage exosomes loaded with cisplatin significantly increased cytotoxicity in drug-resistant ovarian cancer cells (A2780/DDP and A2780 cells) (141), and TNF-α-loaded EV-based vehicles enhanced cancer-targeting under a magnetic field and suppressed tumor growth in murine melanoma subcutaneous models (142). Studies have also shown that exosomes loaded with doxorubicin have the same efficacy as doxorubicin and prevent cardiotoxicity (143). Therefore, EVs are capable of safe and efficient drug delivery and provide a viable alternative to conventional drug delivery in NAFLD.

Synthetic exosome mimics have been fabricated as therapeutic tools for drug delivery and have been reported to have therapeutic effects (144). However, most of these studies are in the laboratory research stage; therefore, it is also necessary to establish reliable assays to assess the therapeutic potential of EVs and further develop them into formal potency tests for promoting the clinical applicability of EVs (145).

## 5 Limitation

The limitations of this study include three aspects below. Firstly, most of our research results are from the laboratory, clinical research data is insufficient. Secondly, current standards of EV detection methods are not consolidated, so it is necessary to further test and standardize the detection technology. Thirdly, current studies are limited to published articles, while ongoing studies are not included. So, it is supposed to track the updated research results.

## 6 Conclusion

EVs contain various biological molecules, including proteins, nucleic acids, and lipids; they play an important role in intercellular communication in various biological processes, including the development and progression of diseases such as NAFLD. EVs participate in different signaling pathways to regulate the initiation and progression of NAFLD. As natural carriers of biological molecules, EVs have potential advantages in the treatment of NAFLD. Circulating EVs have been considered potential diagnostic and prognostic biomarkers fin NAFLD. Exploring the precise mechanism of EVs in NAFLD will help us identify new biomarkers. Research on the mechanisms and clinical applications of EVs in NAFLD is in its initial phase and the applicability of EVs in NAFLD diagnosis and treatment is expected to grow with technological progress.

## Author contributions

YX, J-CC, and WJ are responsible for collecting and sorting the literature and writing the paper. Y-HL, YH, C-HL, EC and HT are responsible for supplementing, revising and improving content. HZ and DW are responsible for guidance and proofreading. All authors contributed to the article and approved the submitted version.
